# Editorial: Dietary strategies for managing hypertension and hypotension: insights and mechanisms

**DOI:** 10.3389/fnut.2025.1648890

**Published:** 2025-07-30

**Authors:** Agnieszka Kujawska, Claire Elizabeth Robertson, Fadi J. Charchar, Nicholas McMahon, José Augusto Simões

**Affiliations:** ^1^Department of Exercise Physiology and Functional Anatomy, Ludwik Rydygier Collegium Medicum in Bydgoszcz Nicolaus Copernicus University in Toruń, Bydgoszcz, Poland; ^2^Cardiology and Cardiac Surgery Department, 10th Military Research Hospital and Polyclinic IPHC in Bydgoszcz, Bydgoszcz, Poland; ^3^School of Life Sciences, University of Westminster, London, United Kingdom; ^4^Health Innovation and Transformation Centre, Federation University Australia, Ballarat, VIC, Australia; ^5^Department of Medicine, Austin Health, University of Melbourne, Melbourne, VIC, Australia; ^6^Department of Cardiovascular Sciences, University of Leicester, Leicester, United Kingdom; ^7^School of Human Movement and Nutrition Sciences, University of Queensland, St. Lucia, QLD, Australia; ^8^CINTESIS - Centre for Research in Health Technologies and Service, Porto, Portugal; ^9^ARS Centro, Coordination of Medical Residency in General Practice/Family Medicine in the Central Zone, Coimbra, Portugal

**Keywords:** hypertension, hypotension, supplements, diet, vitamins, hyperhomocysteinemia, cardiovascular health

## 1 Introduction

The relationship between diet and health has been observed for thousands of years, as evidenced in the works of Galen (1). Galen wrote: “Many of the finest physicians have written about the properties of foods, taking the subject very seriously since it is about the most valuable of any in medicine. (…) But since by holding differing views they have raised suspicions about one another (for they cannot all be speaking the truth!) we must become impartial judges and put what they have said to the test. For without demonstration it is wrong to put one's confidence in one more than the others” (2). Despite the centuries that have passed since Galen's observations, nutrition science still lacks robust data and, consequently, evidence-based consensus in many subfields. With this Research Topic, we aim to extend the current understanding of dietary factors in hypertension (HTN) and cardiovascular health.

## 2 Diet-related factors for hypertension

Diet could be analyzed based on multiple levels, with one of the top layers being dietary patterns. Among the Tibetan population, three predominant dietary patterns were identified (Li X. et al.). Adherence to the “Tsamba-red meat-tuber” pattern was linked to an elevated risk of HTN, whereas the “Rice-vegetable-fruit” and “Dairy products” patterns were associated with a reduced HTN risk. Based on these results, the efficacy of a targeted dietary intervention might be examined among Tibetans residing in the Garze Tibetan Autonomous Prefecture of Sichuan Province (Li et al.).

In the nested case–control study within the Fasa Adult Cohort Study (FACS), involving 975 participants aged 35–70 years, higher antioxidant intake is linked to a lower likelihood of developing HTN, emphasizing the valuable role of antioxidant-rich diets as an adjunctive measure in HTN prevention and management (Firooznia et al.).

A comprehensive study involving 195,250 participants from the UK Biobank cohort investigated the interplay between genetic predisposition and plasma fatty acid (FA) profiles in relation to HTN risk (Lu et al.). Higher plasma levels of polyunsaturated (PUFAs) and n-3 PUFAs were inversely associated with HTN risk, whereas elevated monounsaturated fatty acids (MUFAs) and saturated fatty acids (SFAs) were related to an increased risk (Lu et al.). Notably, a significant additive interaction between genetic risk and plasma FA levels was observed, contributing to a 10%−18% increased HTN risk (Lu et al.).

Another level of diet analysis is based on intake and the level of vitamins and their relationship with health indicators. Wu D. et al. showed that sufficient intake of both Vitamin C and Selenium is linked to a reduced risk of HTN among U.S. women. In a study by Dai et al., participants with higher overall vitamin levels did not exhibit significantly different blood pressure compared to those with lower levels. Nevertheless, plasma 25-hydroxyvitamin D3 demonstrated a modest inverse relationship with systolic blood pressure, whereas elevated α-tocopherol (vitamin E) levels correlated with a slight increase in systolic pressure (Dai et al.). These findings suggest that α-tocopherol may counterbalance the potential protective effect of vitamin D3 on blood pressure, highlighting the complex interplay among fat-soluble vitamins in hypertensive individuals (Dai et al.).

Diet can also be analyzed in terms of specific micronutrient intakes, though such studies are complicated by potential interactions between nutrients. For instance, in comparison to salt restriction, the use of salt substitutes leads to a more pronounced reduction in sodium intake alongside a significant increase in potassium consumption (Wu H. et al.). However, these changes do not translate into superior blood pressure control, particularly among individuals using salt substitutes with 13% potassium chloride content. Notably, only the group exposed to the higher 25% potassium chloride formulation demonstrated a meaningful reduction in systolic blood pressure, underscoring the importance of potassium concentration in the efficacy of salt substitutes for blood pressure management (Wu H. et al.).

Miao et al. sought to clarify the relationship between various human trace elements and essential HTN. Employing two-sample, multivariate, and inverse Mendelian randomization analyses, the investigation focused on 15 trace elements identified through comprehensive database searches (Miao et al.). The analyses revealed a significant link between copper intake and the risk of developing essential HTN. This association was further substantiated by analysis of data from the National Health and Nutrition Examination Survey (NHANES), which revealed higher copper intake to be associated with increased HTN risk (Miao et al.).

It is important to note that hypertension is now recognized as a cluster of disorders with various mechanisms underlying pathology, including genetic, environmental, neurohormonal, renal, vascular, and metabolic factors contributing to its pathogenesis (3). Diet could be used to influence the level of particular risk factors, including homocysteine (Hcy) level (4). The coexistence of hyperhomocysteinemia (HTH) and HTN, termed H-type hypertension, is significantly associated with accelerated renal decline and an increased risk of major adverse cardiovascular and cerebrovascular events in patients with chronic kidney disease not requiring dialysis (Cai et al.).

Among Chinese adults in Hunan Province, the combination of heavy alcohol use, unhealthy diet, and elevated BMI showed the strongest association with HTH, with risk further amplified by the addition of smoking (Li, Wang, Li, Li, Long, et al.). The relationship between the number of unhealthy lifestyle factors and HTH risk followed a J-shaped dose–response curve, underscoring the compounding effect of multiple behaviors (Li, Wang, Li, Li, Long, et al.).

Emerging evidence robustly underscores elevated plasma Hcy levels as a significant independent predictor of cardiometabolic multimorbidity (CMM) (Li, Wang, Li, Li, Wang, et al.). Notably, the synergistic coexistence of diabetes, HTN, and coronary heart disease amplifies this risk, revealing Hcy as a critical modifiable biomarker in CMM pathogenesis (Li, Wang, Li, Li, Wang, et al.).

## 3 Diet-related factors for cardiovascular health

Sato et al. (5) described the relationship between hypoxia-inducible factor 1-alpha (HIF-1α) and cardiovascular health. Guo et al. investigated the relationship between blood concentrations of zinc, iron, and calcium and HIF-1α among individuals residing at different altitudes and belonging to diverse ethnic groups, aiming to deepen understanding of altitude illness mechanisms. Based on serum samples from 400 from Xining and Sanya analysis, significant differences in zinc, calcium, and HIF-1α levels were observed between low- and high-altitude populations, while iron levels remained consistent (Guo et al.). Variations in microelements and HIF-1α blood levels were found to be related to altitude and ethnicity, potentially influencing the onset and progression of altitude-related illnesses (Guo et al.).

A comprehensive analysis of over 460,000 UK Biobank participants examined the associations between hydration sources, including water, coffee, and tea, with cardiovascular disease (CVD) risk over a median follow-up of 8.7 years (Ke et al.). Higher water intake was related to a reduced risk of heart failure, coronary heart disease, and stroke in both men and women. Conversely, high consumption of coffee (six or more cups daily) and tea was associated with an elevated risk of these cardiovascular outcomes (Ke et al.). In addition, an excessive coffee and tea intake appeared to diminish the protective effects of water consumption on CVD risk (Ke et al.).
